# Attitudes of a Hemato-Oncology Team Toward Referring Patients to Palliative Care: A Qualitative Study

**DOI:** 10.1177/10966218251366067

**Published:** 2025-08-28

**Authors:** Kimberly Botero, Cristian Rendón, Alicia Bernal-García, Diana Borda

**Affiliations:** ^1^ Pain and Palliative Care Medicine, Fundación Universitaria Sanitas, Bogotá, Colombia.; ^2^ Palliative Medicine Program, Clínica Universitaria Colombia, Bogotá, Colombia.

**Keywords:** hematological malignancies, hematology, leukemia, lymphoma, multiple myeloma, palliative care

## Abstract

**Objective:**

International literature suggests that patients with hematological diseases are frequently referred to palliative care (PC) at a late stage. This study aims to explore the attitudes of a hemato-oncology care team toward referring patients to the PC in a fourth-level hospital in Bogotá, Distrito Capital.

**Methods:**

This exploratory qualitative study was conducted through in-person focus groups between May 2024 and October 2024 at Clínica Universitaria Colombia in Bogotá, Colombia. Data were analyzed thematically using framework analysis, an analytical approach that structures qualitative data around predefined themes aligned with the research question.

**Results:**

This study identified key factors influencing the referral of patients with hematological malignancies to PC. These include symptom management, interdisciplinary collaboration, and perceived barriers such as misconceptions about PC, emotional attachment, and limited formal training among health care professionals.

**Conclusion:**

While the hematology team acknowledges the value and benefits of PC, barriers such as prognostic uncertainty, emotional attachment, and insufficient training continue to hinder its early integration into the management of patients with hematological malignancies.

## Introduction

The World Health Organization (WHO) defines palliative care (PC) as an approach that enhances quality of life for patients with life-threatening illnesses and their families.^1–3^ Early integration has been shown to improve symptom management and may even extend survival.^1,3,4^ In Colombia, Law 1733 of 2014 regulates PC, mandating comprehensive management for patients with chronic, degenerative, and incurable diseases.^5^ Despite this legal framework, Colombia continues to face a high burden of unmet PC needs. These are exacerbated by marked regional disparities, reflecting persistent inequities in access to specialized health services. Addressing this gap requires urgent action to strengthen the national PC infrastructure, expand service coverage, and ensure the integration of PC across all levels of the health care system.^6^

In this context, Bogotá—the capital city—reports one of the highest PC service availability rates in the country, with two PC services per 100,000 inhabitants.^6^ However, this remains insufficient to meet current demand, highlighting the need to increase capacity and improve access even in regions with relatively better service provision.^6^

A particularly concerning issue is the lack of local data on PC referrals for patients with hematological malignancies. This limits the ability to evaluate and enhance integration within hematology services.^6^ This gap is especially relevant given that patients with hematological neoplasms are referred to PC less frequently than those with solid tumors, despite experiencing a comparable burden of symptoms.^7–9^ Several factors contribute to this disparity. The unpredictable clinical trajectory of hematological diseases often complicates timely referral, resulting in delayed PC involvement until the disease reaches an advanced stage.^10–12^ This underscores the importance of interdisciplinary collaboration to strengthen patient-centered care.^10,13^

Despite global recognition of the role PC plays in enhancing quality of life, only 14% of the 40 million people who need it receive adequate support—particularly in low- and middle-income countries.^3,14^ Hematological malignancies require specialized PC; however, unclear prognostic markers often hinder early integration.^13,15,16^ Additionally, differences in perspective between hematologists and oncologists further emphasize the need to examine referral practices.^17–19^

Therefore, this study explores the attitudes and practices of a hemato-oncology team in a tertiary hospital in Bogotá, offering insights into current referral patterns and identifying potential barriers to early PC involvement.

## Methods

This study employed a qualitative, exploratory design to examine health care professionals’ experiences in referring hemato-oncological patients to PC. Four in-person focus groups were conducted with members of the hemato-oncologist team at Clínica Universitaria Colombia in Bogotá, Distrito Capital, between July and December 2024. A thematic analysis was carried out to identify key themes emerging from the discussions.^20^

Participants were selected through purposive sampling and included 13 professionals with over 1 year of experience: 8 hemato-oncologists, 2 head nurses, 1 general physician, 1 psychologist, and 1 social worker. Each session was facilitated by a moderator and accompanied and accompanied by a notetaker. After obtaining informed consent, the discussions followed a semistructured guide that explored referral factors, barriers, facilitators, the impact on patients and families, and training needs. The guide was reviewed and validated by a PC specialist and a psychologist. Sessions lasted 40–50 minutes and included four to five participants each. All sessions were audio-recorded and transcribed verbatim. Thematic data analysis included initial categorization, coding, and researcher triangulation to ensure rigor and reliability.

The study complied with national ethical guidelines (Resolution 8430 of 1993)^21^ and was classified as minimal risk by the Fundación Universitaria Sanitas ethics committee given the exploration of sensitive behavioral aspects. An expert psychologist was present during the focus groups to provide emotional support and crisis containment if needed. Informed consent was obtained from all participants, and confidentiality was strictly maintained; all data were securely handled by the research team.

## Results

This section outlines the thematic analysis and coding process. A series of central themes and categories were identified, each presented below with its corresponding definition and findings. The full set of categories is summarized in Table 1. Deductive categories reflected team attitudes and referral processes, as predefined by the research questions. Additionally, an inductive category—emotions during referral—emerged through open coding. The findings are organized and described below according to these categories.

**Table 1.** A Priori and Emergent Categories

A priori categories	Emergent category
Definition of palliative care	Perceived emotions during the referral to palliative care
Key factors in the referral process	
Barriers and challenges in the process	
Facilitators and support in referral	
Impact of palliative care on the patient and family	
Potential improvements in the referral process	
Training and education of the hemato-oncology team	

### Definition of PC

Participants described PC as a comprehensive specialty that goes beyond symptom management to address emotional, social, and spiritual needs. They emphasized its role in supporting patients and families throughout the course of chronic illness—not just at the end of life—promoting comfort, dignity, and quality of life at any stage. PC was viewed as an essential complement to clinical care, offering a multidisciplinary approach that helps align treatment goals with patients’ values and preferences: “It is like our ally … they are our right hand.” (Hematologist). Table 2 shows some representative quotes that reflect participants’ conceptualization of PC.

**Table 2.** Conceptualization of Palliative Care from a Hemato-Oncology Team

Mentioned concepts	Relevant aspects	Representative quotes
Integrated approach to care	Multidisciplinary approach	“It is that specialty that has the ability to provide support, assistance and to help patients in the management of all the things that surround them.” “It is the assistance of the whole team.”*“*It helps to face the whole coping or path that leads to death from all psychological, social and medical points of view.”
	Complement in clinical intervention	“It is in charge of managing the symptoms that come from chronic diseases, both oncologic and non-oncologic, chronic and acute.” “It must be a specialty that tries to manage in a comprehensive manner all the needs that I, as a clinician, am sometimes unable to meet.” “It is the assistance of every person in the process that leads to a chronic disease that is not necessarily curable.” “It is not simply limited to pain alone, it also encompasses many other things that one does not know about.”
	Coordination among specialties	“I think it’s that, a complement for us, because of all the characteristics of hemato-oncologic diseases.” “Our right hand in helping in many things from a diagnosis to a final stage of life.”
Patient and family support	Improved acceptance and quality of life	“It is a tool that allows us to provide patients with a better acceptance of their disease and their family.” “It is the patient’s hope when the therapeutic objective is not curative and a well-being is sought.” “It is the assistance, determining how to improve the patient’s quality of life and symptoms, regardless of whether it is pain or not.”
	Attention to the family as part of care	“…and that guarantees the patients’ quality of life and family support.” “It also encompasses the family, the patient’s entire environment at that moment. All the social and psychological aspects.”
Assistance during the course of illness	Occurs at an early stage or in chronic disease	“For me it is the assistance of every person in the process that leads to having a chronic non-curable disease.” “To sensitize them a little bit to the fact that not everything is the end of life, but also wellbeing, pain management, to be able to provide a better quality environment, to be able to provide an environment at home, to see what else can be done.”
	Does not represent therapeutic abandonment	“It doesn’t mean that nothing is being done; there is a lot to be done.” “Fundamental pillar in the treatment.”

### Facilitators in the referral of patients with hematological malignancies to PC

The hemato-oncology team identified three primary factors influencing referrals to PC: (1) the need for specialized symptom management; (2) the patient’s acceptance of the disease trajectory—particularly during critical moments such as communication of bad news and the establishment of goals of care; and (3) the early involvement of interdisciplinary support aimed at addressing the complex needs of both patients and their families.

First, PC was viewed as essential for symptom control, with particular emphasis on pain management, considered a central concern. Second, the team underscored the role of PC in promoting acceptance of the disease by aligning clinical decisions with patients’ individual goals. This approach fosters greater acceptance among both patients and families. As one hematologist explained: “It allows us to provide patients and their families with greater acceptance of everything that the disease entails.” By supporting a patient-centered transition, PC helps ensure that treatment decisions reflect personal values and are not perceived as abandonment but as care tailored to individual needs and expectations.

Last, participants emphasize the value of integrated support beginning at the time of diagnosis, facilitated through the involvement of a multidisciplinary team. This coordinated model enhances communication between teams, eases the delivery of difficult news, and promotes a compassionate, humanized approach to care: “When the support is good, you can tell. The patient does well, they feel at peace” (Hematologist).

### Barriers and challenges in referring patients with hematological malignancies to PC

**FIG. 1.**
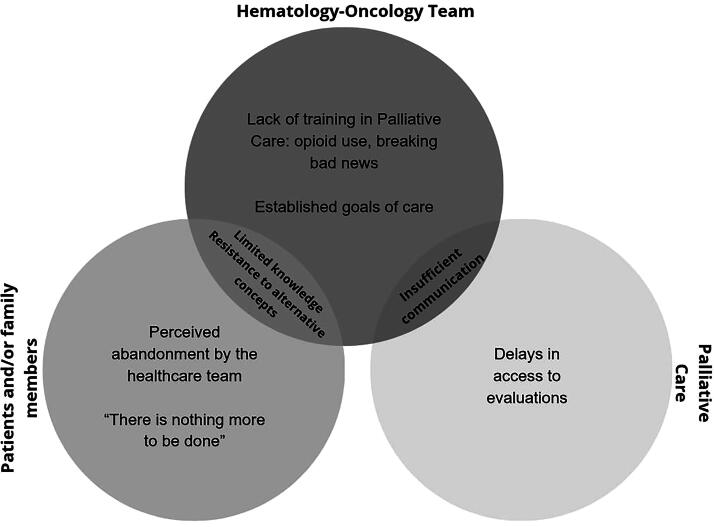
Perceived barriers in patients, the hemato-oncology team, and the palliative care team in the referral process.

Participants identified several barriers to PC referral (Fig. 1). A key challenge is the persistent misconception that PC is equivalent to abandonment or euthanasia—a belief held by both patients and some clinicians. As one hematologist remarked: “They believe there is nothing more to be done … many think it is synonymous with euthanasia.” This stigma, coupled with the strong physician–patient bond, can limit openness to PC involvement: “Patients experience long hospitalizations under their primary physician’s care, making it difficult to accept guidance from someone else.”

Additional barriers include insufficient training in PC—especially in opioid management, communication skills, and family support—as well as suboptimal coordination between the PC and hemato-oncology teams. As one hematologist noted: “We may end up saying different things, making it seem as if our approaches are mutually exclusive.”

To address these challenges, participants recommended implementing structured interdisciplinary meetings and joint case discussions. Access-related issues were also highlighted, such as delays in scheduling PC appointments, with patients often facing difficulties securing timely consultations.

### Impact on the patient and family when referred to PC

The hemato-oncology team observes that referrals to PC often trigger initial fear and resistance among patients. Statements such as “Why are they sending me there if I’m not that bad?” reflect the lingering stigma that PC is only offered when “there is nothing more to be done.”

Over time, however, these perceptions often shift. Effective symptom control—particularly pain management—enhances quality of life and helps reshape how patients view PC. Many express gratitude not only for the medical care but also for the psychosocial support they receive, which provides emotional relief and fosters a more positive understanding of PC as an integral part of their treatment journey.

### Training and education of the hemato-oncology team in PC

Among the eight hematologists interviewed, only two reported having received formal PC training during their postgraduate studies—one in Brazil and one in Colombia. Both head nurses also completed postgraduate training in PC, while the remaining participants reported no formal education in the field. Although some had exposure through rotations or isolated courses, many acknowledged gaps in key areas such as pain management and symptom control. Several expressed uncertainty in prescribing opioids or using transdermal patches. As one general practitioner noted: “If you asked me when to prescribe a lidocaine patch, I honestly wouldn’t know.” Despite these limitations, participants demonstrated a strong interest in acquiring practical skills, particularly in opioid management. One hematologist shared: “Converting IV opioids to oral—those are aspects we could manage ourselves with some training.” Suggested strategies to address these gaps included lectures, workshops, and mandatory PC rotations during postgraduate training.

### Emotions perceived during referral to PC

A notable finding was the emotional burden experienced by the hematology team, marked by strong attachment and a deep sense of responsibility toward their patients. The physician–patient relationship was described as “maternalistic,” reflecting the strong bonds formed during prolonged hospitalizations. As one hematologist shared: “It’s like my own child who has been here, under my care, for four months.” This emotional connection can complicate decision making and potentially delay referrals to PC. “That maternal instinct… sometimes leads us to excesses,” noted another hematologist. Patient deaths were described as deeply painful experiences, underscoring the need for institutional support to help health care professionals manage grief.

## Discussion

Based on the findings of this study, three key themes emerged: the importance of early integration of PC in patients with hematological malignancies; the development of strong emotional bonds between clinicians and patients—commonly referred to in the literature as medical maternalism; and the need to strengthen inter-team communication and collaboration.

**FIG. 2.**
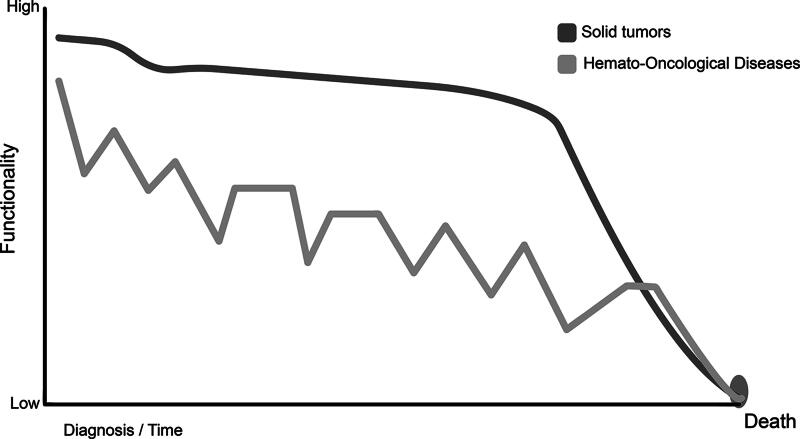
Possible scenarios and trajectories in hemato-oncological diseases versus Solid tumors

First, while early integration of PC in hemato-oncology offers multiple benefits,^22^ it also presents distinct challenges due to the unpredictable and fluctuating trajectory of hematological malignancies. These trajectories differ markedly from those of solid tumors.^23^ Whereas patients with solid tumors typically follow a more linear decline that facilitates clearer transitions to PC, hemato-oncological patients often experience alternating periods of remission and relapse^24^ (Fig. 2). This fluctuation, combined with the potential for response to high-intensity treatments even at advanced stages, sustains a clinical culture that emphasizes the possibility of recovery and complicates decisions around the discontinuation of aggressive treatment.^17,25,26^

The availability of novel therapies—including transplants, immunotherapies, and clinical trials—adds to the complexity of care by continually introducing new treatment options. While these innovations are promising, they may inadvertently delay the integration of PC and prolong interventions that can become nonbeneficial.^9,27^

**FIG. 3.**
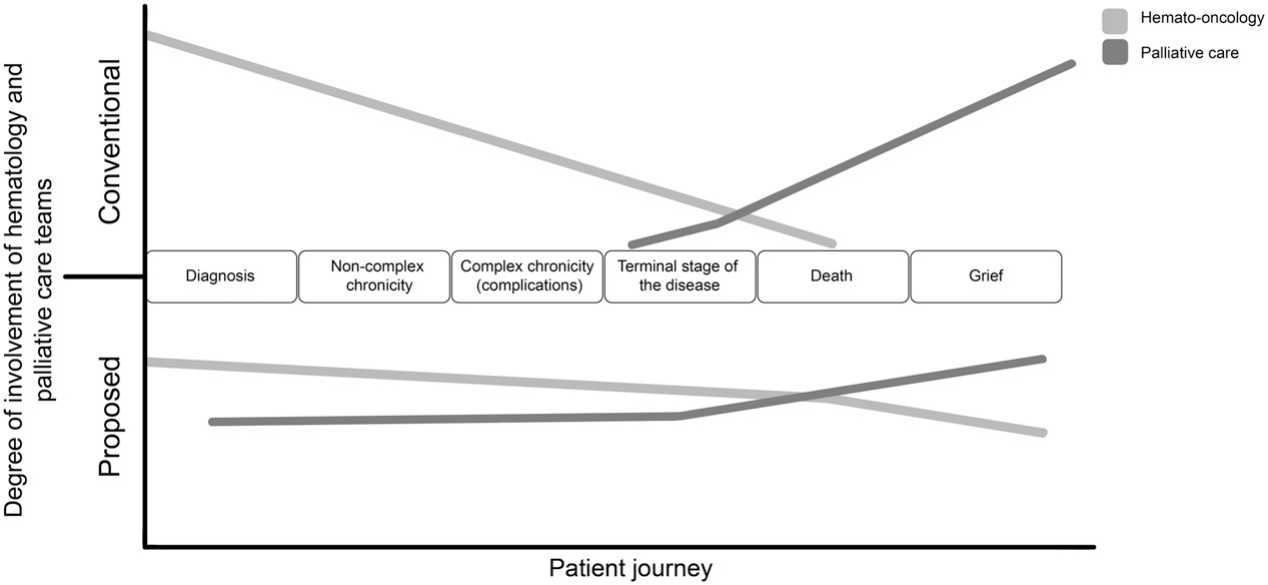
Comparison of the conventional and proposed models for intervention and integration of hemato-oncology and palliative care specialties.

Despite these challenges, participants in this study acknowledged the value of integrating PC earlier in the disease trajectory. As illustrated in Figure 3, the conventional model tends to delay PC until the terminal phase. In contrast, the proposed model promotes a gradual and sustained collaboration between hemato-oncology and PC from earlier stages. This integrated approach is designed to enhance quality of life, optimize symptom control, and provide comprehensive support—including bereavement care—for both patients and their families.

Second, the formation of emotional bonds between hematologists and their patients emerged as a recurring theme. These relationships were often described by participants as a form of maternalism, distinct from traditional paternalism. Instead of making unilateral decisions, maternalism is characterized by guiding patients through a relationship grounded in emotional closeness and mutual trust.^28,29^ In the hemato-oncological setting—where lengthy hospitalizations and extended treatment courses are common—these emotional connections tend to deepen. As one clinician shared: “They are like my child; they have been under my wings for four months.” However, such closeness can complicate the transition to PC. As one participant acknowledged: “Sometimes we don’t know how to stop,” capturing the tension between continuing aggressive treatment and prioritizing the patient’s comfort.

Interestingly, although emotional engagement was clearly present, participants did not explicitly articulate or label their feelings. Instead, they expressed their affective experiences through stories and personal reflections. This pattern may indicate a difficulty in recognizing or verbalizing their own emotional responses—a dynamic that could influence clinical decision making, particularly when strong emotional involvement delays timely shifts in the therapeutic approach.

Last, the need to improve interdisciplinary and transdisciplinary communication, as well as to incorporate PC training into hematology education, emerged as critical elements for optimizing care. Participants identified key barriers, such as fragmented communication, lack of shared goals, and limited opportunities for joint decision making. Enhancing communication across teams is therefore essential—not only to align treatment objectives but also to ensure that patients receive holistic, coordinated, and timely care aligned with their values and needs throughout the illness trajectory.

The complexity of hemato-oncological cases and symptom management underscores the importance of PC training and mandatory rotations that can foster more effective collaboration between specialists.^30^ The integration of PC from earlier stages has the potential to improve outcomes, yet the challenges posed by disease complexity and fragmented care models remain significant.^27,31^ Overcoming these challenges is key to delivering coordinated, value-based care throughout the disease trajectory.

Embedding shared decision making within a collaborative inter-team model—linking hematology and PC through structured tools and ongoing dialogue—helps ensure that care remains aligned with patients’ evolving values.^32^ Evidence suggests that these tools improve patient understanding, satisfaction, and reduce decisional conflict in hematological malignancies. This approach reframes PC not as a last resort but as an integral resource that should be introduced from the early stages of treatment.^31^

## Conclusions

The integration of PC in hemato-oncology encounters several barriers, including difficulties in predicting prognosis, the perception of disease reversibility, and the strong emotional bonds formed between clinicians and patients. While specialists recognize its value in symptom management and comprehensive support, implementation remains delayed. Contributing factors include a lack of specific training and limited interaction between clinical teams, which further complicate the transition.

Fostering shared decision making among the hemato-oncology team, patients, and their families can promote greater understanding and earlier acceptance of PC as an integral component of treatment. Enhancing education in pain management and communication—combined with closer collaboration between hemato-oncology and PC teams—can strengthen the quality of care and significantly improve the experience for patients and their families.
